# The association between urinary incontinence and suicidal ideation: Findings from the National Health and Nutrition Examination Survey

**DOI:** 10.1371/journal.pone.0301553

**Published:** 2024-05-23

**Authors:** Ting Pan, Zhiguo Zhang, Tiantian He, Chongyang Zhang, Junjie Liang, Xinru Wang, Xueshi Di, Yuying Hong, Peng Bai

**Affiliations:** 1 Department of Acupuncture, Beijing University of Chinese Medicine Third Affiliated Hospital, Beijing, 100029, China; 2 China Academy of Chinese Medical Sciences Institute of Basic Theory in Chinese Medicine, Beijing, 100700, China; Chiba Daigaku, JAPAN

## Abstract

**Background:**

Urinary incontinence (UI) might be linked to suicidal ideation, but we do not yet have all the relevant details. This study aimed to dig deeper into the connection between UI and suicidal ideation using data from the National Health and Nutrition Examination Survey (NHANES).

**Methods:**

We examined 31,891 participants aged ≥ 20 years from NHANES 2005–2018 who provided complete information. We used standardized surveys to check for UI and signs of suicidal ideation. To better understand this relationship, we used statistical tools such as multivariable logistic regression, subgroup analysis, and sensitivity analyses.

**Results:**

Among the 31,891 participants, 28.9% reported UI and 10.7% reported suicidal ideation. Those with UI exhibited a significantly greater incidence of suicidal ideation (15.5%) than did those without UI (8.8%, *P* < 0.001). After adjusting for various factors, including age, sex, marital status, socioeconomic status, educational level, lifestyle factors, and chronic comorbidities, UI remained significantly associated with suicidal ideation (OR:1.54, 95% CI = 1.39–1.7, P < 0.001). Among all types of UI, MUI participants were more likely to experience suicidal ideation. Compared with no UI, higher odds of suicidal ideation suffered from MUI (OR:2.11, 95%CI:1.83–2.44, *P* < 0.001), SUI (OR:1.4, 95%CI:1.19–1.65, *P* < 0.001), UUI(OR:1.37,95%CI:1.16–1.62, *P* < 0.001) after full adjustment. With the exception of individuals living with a partner, the remaining subgroups exhibited a positive correlation between urinary incontinence and suicidal ideation, considering that factors such as age, sex, and prevalent comorbidities such as hypertension, depression, and diabetes did not reveal any statistically significant interactions (all *P* > 0.05). Sensitivity analyses, incorporating imputed missing covariates, did not substantially alter the results (OR: 1.53, 95% CI: 1.4–1.68, *P* < 0.001).

**Conclusion:**

Urinary incontinence may correlate with increased suicidal ideation risk, priority screening for suicidal ideation and timely intervention are essential for individuals with urinary incontinence, but prospective studies are needed to verify the results.

## Introduction

Suicide represents a critical global health concern, with estimates indicating 16 million attempts and 800,000 deaths annually [1]. In the United States alone, over 30,000 individuals die by suicide each year [2]. Suicide prevention constitutes a pivotal component of the WHO’s strategic plan aimed at reducing global suicide rates by one-third in each country by the year 2030 [3]. Empirical evidence underscores a robust correlation among suicide, suicide attempts, and suicidal ideation [4,5]. Suicidal ideation frequently serves as a significant precursor to both suicide attempts and completed suicides [6–8]. Estimates indicate that approximately 60 percent of individuals experiencing suicidal ideation progress to a suicide attempt within one year [9]. Moreover, a recent meta-analysis has identified an elevated risk of suicide among individuals with suicidal ideation [6]. Identifying risk factors for suicidal ideation is essential in this landscape. Notably, a correlation is observed between urinary incontinence (UI) and increased prevalence of mental health issues like anxiety and depression, yet the direct relationship between UI and suicidal ideation is not well established [10–13].

Defined as involuntary urine leakage, UI is a widespread issue causing significant embarrassment and distress worldwide [14], leading to substantial societal and individual burdens [15]. Its primary variants—Stress Urinary Incontinence (SUI), Urgency Urinary Incontinence (UUI), and Mixed Urinary Incontinence (MUI)—impart a profound impact on individuals’ psychosocial well-being. In the Korean population, elderly women experiencing urinary incontinence face a 2 fold and 1.5-fold increased risk of stress and depression, respectively, compared to those without urinary incontinence [12]. A Norwegian study, spanning a decade, revealed a 45 percent increase in mild depression and a 26 percent rise in anxiety among women with urinary incontinence [16]. Additionally, individuals with urinary incontinence exhibited heightened levels of loneliness, diminished stress resilience, and reduced self-esteem, with loneliness being 1.51 times more prevalent in those with UI compared to their counterparts without UI [17]. Notably, the Korean female urinary incontinence population displayed significantly elevated levels of depression and stress, coupled with lower levels of self-esteem [10]. Emotional disorders associated with urinary incontinence, including embarrassment, social isolation, and diminished self-esteem, were found to be significantly correlated with depression [18,19]. Depression, a well-established precursor to suicidal ideation, further underscores the intricate emotional challenges faced by individuals grappling with urinary incontinence [20,21]. Despite these implications, focused research on UI’s role in suicidal ideation within the U.S. population remains lacking.

This study aims to fill this gap, utilizing the comprehensive data from the 2005–2018 National Health and Nutrition Examination Survey (NHANES). It seeks to explore the association between urinary incontinence and suicidal ideation among American adults and ascertain whether this association exhibits consistency across varied demographics and co-existing conditions, such as age, gender, and prevalent comorbidities including diabetes, depression, and hypertension. By establishing a clearer understanding of these relationships, the study aims to contribute significantly to the nuanced public health strategies needed to address and mitigate suicide risks.

## Methods

### Study design and eligibility criteria

The NHANES (https://www.cdc.gov/nchs/nhanes/), conducted by the National Center for Health Statistics (NCHS), is a nationally representative survey employing stratified, multistage probability cluster sampling methodologies to comprehensively evaluate the health status of the noninstitutionalized population in the U.S. This cross-sectional study analyzed deidentified data from the 2005–2018 NHANES cycles for participants aged ≥ 20. Approval for the NHANES study protocol was obtained from the NCHS research ethics review board, and participants provided written informed consent.The use of publicly available deidentified data and the need to provide consent were waived. The study adhered to the STROBE reporting guidelines.

**Fig 1:**
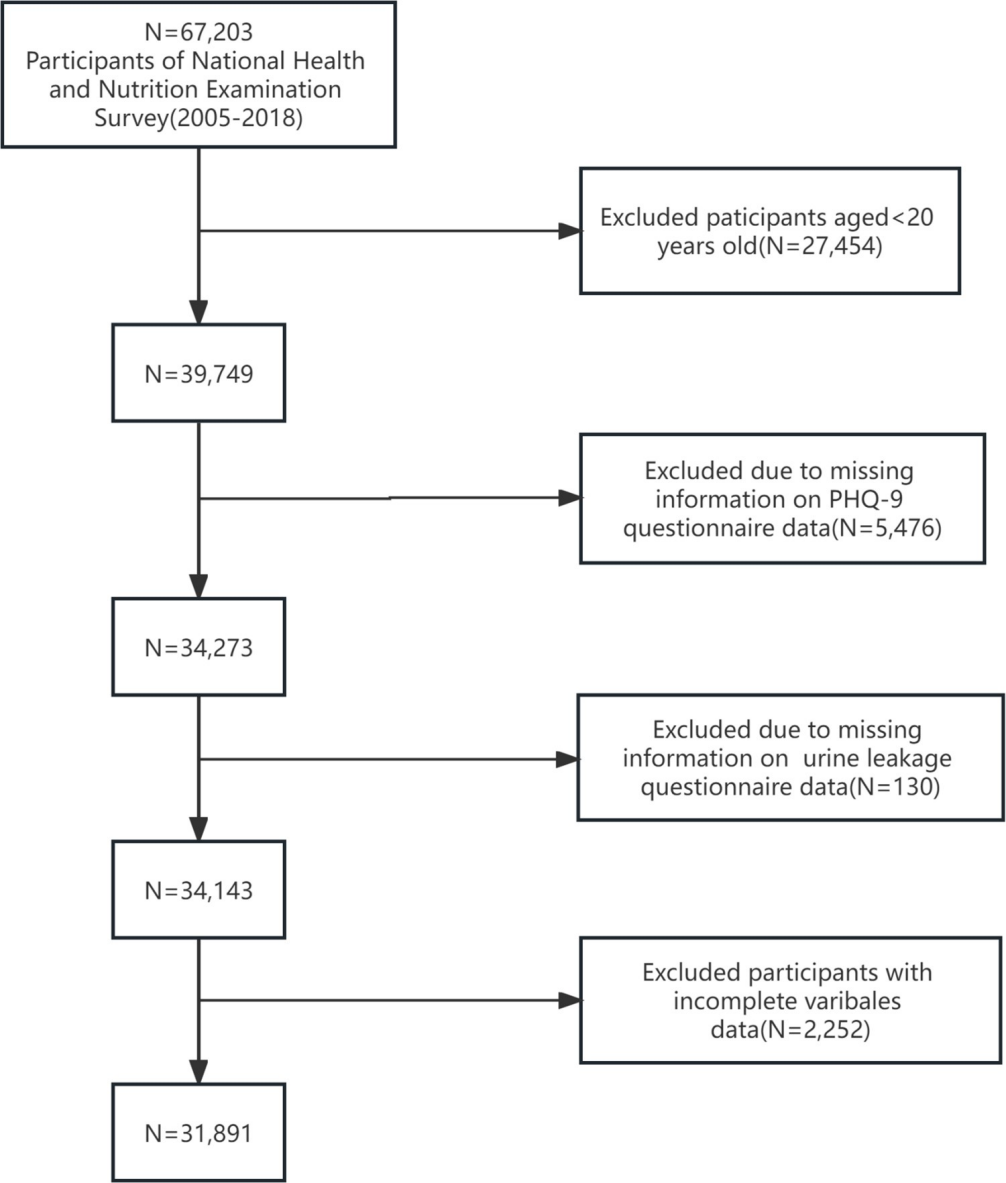
Study flowchart.

Our analyses were based on data collected from participants during seven 2-year NHANES cycles (2005–2018). In this study, to specifically focus on participants experiencing suicidal ideation, we established exclusion criteria that included individuals under 20 years old, those with missing information on suicidal ideation or urinary incontinence (UI), and incomplete data regarding risk behaviors, associated comorbid conditions, or demographic details. Given the substantial size of our sample, instances of missing values for various covariates were minimal, accounting for less than 5% of the total data, with alcohol intake data missing in only 3.9% of the patients. Consequently, we opted for direct deletion of these instances. The process of participant selection is detailed in Fig 1, leading to a final analytical data of 31,891 participants.

### Definition of suicidal ideation

The Patient Health Questionnaire-9 (PHQ-9) is a diagnostic instrument comprising nine items that was designed to assess the presence of depressive symptoms experienced by an individual over the preceding two-week period. Among the general population, this tool is recognized for its substantial validity and reliability in screening for depression [22]. Suicidal ideation was assessed by item 9 of the PHQ-9 as the outcome variable. This variable has been previously validated to identify outpatients with an increased risk of suicide attempt or death [23]. Participants were identified as having suicidal ideation based on their affirmative responses to the following question: "In the past two weeks, have you been troubled by any of the following issues? feeling that you would be better off dead or of hurting yourself in some day?" For the purpose of analysis, the responses were classified into two categories: absence of suicidal thoughts (No) and presence of suicidal thoughts at any frequency (Yes) [24–27].

### Assessment of UI

The evaluation of UI was conducted through analysis of responses from a kidney condition survey. The determination of UI presence was based on answers to the following query: "How often do have urinary leakage?" Participants who indicated "Never" were categorized as not having UI, while any other response indicated the presence of UI. Further classification of UI types was guided by specific questions within the questionnaire: SUI was identified if participants confirmed leakage during physical activity, and UUI was diagnosed when individuals reported involuntary urination before reaching the toilet. Those who responded affirmatively to both criteria were categorized as having MUI. Patients who did not fit into these categories were considered to have other types of UI [28,29].

### Covariates

The statistical model included several covariates, including age (years), sex, race/ethnicity, marital status, poverty income ratio (PIR), educational level, smoking status, alcohol intake, physical activity (converted to metabolic equivalent (MET) minutes), body mass index (BMI), hypertension, diabetes, cardiovascular disease (CVD) history, arthritis, pulmonary disease (emphysema, asthma, or chronic bronchitis), liver disease, cancer, and depression, which were considered potential confounding factors.

Age was categorized into two groups (<60, ≥60), BMI into three groups (<25, 25–30, ≥30), and PIR into three groups (≤1.3, 1.3–3.5, ≥3.5). Self-reported race and ethnicity were classified into five categories: Mexican American, non-Hispanic White, non-Hispanic Black, and other Hispanic. Educational level had three categories: less than high school, high school or equivalent, and above high school. Marital status was classified as never married, married or living with a partner, or other (including widowed, divorced, or separated).

Smoking status was divided into current, former, and never smokers, with "never smokers" having smoked fewer than 100 cigarettes in life, "former smokers" having smoked more than 100 cigarettes but not currently smoked, and "current smokers" having smoked more than 100 cigarettes and currently smoking some days or every day [30].

The alcohol intake categories used were defined as follows: "never" (consumed fewer than 12 drinks in their lifetime); "former" (consumed at least 12 drinks in one year but did not drink in the last year or did not drink in the last year but had at least 12 drinks in their lifetime); "current heavy alcohol use" (consumed at least 3 drinks per day for females, at least 4 drinks per day for males, or engaged in binge drinking on 5 or more days per month); "current moderate alcohol use" (consumed at least 2 drinks per day for females, at least 3 drinks per day for males, or engaged in binge drinking on at least 2 days per month); and "current mild alcohol use" (consumed at most 1 drink per day for females, at most 2 drinks per day for males) [31].

Physical activity referred to the time spent on activities such as walking, biking, work, and recreational activities during the week [32,33]. Hypertension was ascertained through the presence of a mean systolic blood pressure ≥140 mmHg and/or diastolic blood pressure ≥90 mmHg, self-reported diagnosis, or the utilization of antihypertensive medication. Diabetes status was identified based on criteria that encompassed a doctor’s diagnosis, glycohemoglobin (HbA1c) levels ≥ 6.5%, fasting glucose levels ≥ 7.0 mmol/L, random/two-hour oral glucose tolerance test (OGTT) blood glucose levels ≥ 11.1 mmol/L, or the use of diabetes medication/insulin [30,32,33].

The Medical Conditions Questionnaire included inquiries regarding associated comorbidities such as liver disease, a history of CVD history, arthritis, pulmonary disease, and cancer [24].

With reference to previous research on suicidal ideation in the NHANES database [24,25,27], depression was assessed through the PHQ-8 score, with a potential range of 0–24. The PHQ-9 item related to suicidal ideation was excluded, and a score of 10 was used as the cutoff for inclusion in the depression group [34].

### Statistical analyses

A comprehensive descriptive analysis was conducted for all participants; continuous data are presented as the mean and standard deviation (SD), while categorical variables are expressed as percentages (%). Categorical variables were assessed using the chi-square test, and continuous variables (such as age) were analyzed through t tests.

All the statistical data, including the 95% confidence interval (CI) and odds ratios (OR), are presented. To investigate the link between urinary incontinence (UI) and depression, we developed four logistic regression models. Model I made no covariate adjustments. Model II was adjusted for demographic and lifestyle variables such as age, sex, marital status, and health behaviors. Model III included adjustments for chronic conditions such as hypertension and diabetes. Model IV was further adjusted for depression. Subgroup and interaction analyses explored variations in UI and suicidal ideation across demographic variables, health-related factors, and chronic conditions. Sensitivity analyses were performed for robust results using multiple imputations for missing values under 5% for all variables.

The studies involving human participants were reviewed and approved by the institutional review board of the National Center for Health Statistics, CDC. The patients/participants provided their written informed consent to participate in this study [35].

All analyses were performed using R software and Free Statistics software version 1.7. Statistical significance was determined through a two-tailed test, with a *P* value threshold set at less than 0.05.

## Results

### Characteristics of participants in this study

The final analysis included 31,891 participants from the 2005–2018 NHANES cycles, with 9,220 reporting urinary incontinence (UI), reflecting a prevalence rate of 28.9%. Participant demographics, lifestyle factors, and comorbidities are detailed in Table 1. MUI emerged as the predominant UI type, constituting 27.9% of the patients, followed by SUI at 27.8% and UUI at 25.4%. Notably, UI patients were more likely to be older, male, have a higher BMI, be of Mexican American ethnicity, and be never married. Individuals in these groups also tended to be nonsmokers, drinkers, engage in less physical activity, and have a higher incidence of comorbidities such as hypertension, diabetes, arthritis, pulmonary disease, CVD history, liver disease, and cancer. Among these individuals, suicidal ideation was present in 3,422(10.7%) participants. Significantly, UI patients had a greater likelihood of suicidal ideation (15.5%) than did patients without UI (8.8%, P < 0.001). Additionally, a significant increase in depression rates was observed in UI patients (14%) compared with those without UI (6.2%, P < 0.001).

**Table 1:** Demographic and clinic characteristics according to suicidal ideation NHANES 2005–2018.

Variables	Total (n = 31891)	No UI(n = 22671)	UI (n = 9220)	P-value
**Age, Mean ± SD**	49.5 ± 17.6	46.8 ± 17.4	56.4 ± 16.2	< 0.001
**Sex, n (%)**				< 0.001
Female	15925 (49.9)	13393 (59.1)	2532 (27.5)	
Male	15966 (50.1)	9278 (40.9)	6688 (72.5)	
**BMI(kg/m2), Mean ± SD**	29.2 ± 7.0	28.6 ± 6.6	30.7 ± 7.7	< 0.001
**Race, n (%)**				< 0.001
Mexican American	13720 (43.0)	9149 (40.4)	4571 (49.6)	
Non-Hispanic Black	6840 (21.4)	4990 (22)	1850 (20.1)	
Non-Hispanic White	4962 (15.6)	3625 (16)	1337 (14.5)	
Other Hispanic	3048 (9.6)	2265 (10)	783 (8.5)	
Other Race	3321 (10.4)	2642 (11.7)	679 (7.4)	
**Marital status, n (%)**				< 0.001
Married	16412 (51.5)	11634 (51.3)	4778 (51.8)	
Never married	5825 (18.3)	4728 (20.9)	1097 (11.9)	
Living with a partner	2602 (8.2)	2033 (9)	569 (6.2)	
Other	7052 (22.1)	4276 (18.9)	2776 (30.1)	
**PIR, n (%)**				0.233
< = 1.3	9013 (30.8)	6423 (30.9)	2590 (30.5)	
1.3–3.50	11087 (37.9)	7809 (37.6)	3278 (38.6)	
>3.50	9165 (31.3)	6548 (31.5)	2617 (30.8)	
**Education level, n (%)**				0.893
Less than high school	7650 (24.0)	5445 (24)	2205 (23.9)	
High school or equivalent	7329 (23.0)	5194 (22.9)	2135 (23.2)	
Above high school	16912 (53.0)	12032 (53.1)	4880 (52.9)	
**Smoking status, n (%)**				< 0.001
Former	17486 (54.8)	12515 (55.2)	4971 (53.9)	
Never	7732 (24.2)	5271 (23.2)	2461 (26.7)	
Current	6673 (20.9)	4885 (21.5)	1788 (19.4)	
**Alcohol intake, n (%)**				< 0.001
Former	4504 (14.1)	3096 (13.7)	1408 (15.3)	
Heavy	5202 (16.3)	3434 (15.1)	1768 (19.2)	
Mild	10761 (33.7)	7634 (33.7)	3127 (33.9)	
Moderate	4930 (15.5)	3425 (15.1)	1505 (16.3)	
Never	6494 (20.4)	5082 (22.4)	1412 (15.3)	
**MET, n (%)**				< 0.001
<500	13121 (41.1)	8645 (38.1)	4476 (48.5)	
> = 500	18770 (58.9)	14026 (61.9)	4744 (51.5)	
**Hypertension, n (%)**				< 0.001
No	18313 (57.4)	14070 (62.1)	4243 (46)	
Yes	13578 (42.6)	8601 (37.9)	4977 (54)	
**Diabetes, n (%)**				< 0.001
No	25982 (81.5)	19095 (84.2)	6887 (74.7)	
Yes	5909 (18.5)	3576 (15.8)	2333 (25.3)	
**Arthritis, n (%)**				< 0.001
No	23173 (72.7)	17761 (78.3)	5412 (58.7)	
Yes	8718 (27.3)	4910 (21.7)	3808 (41.3)	
**Pulmonary disease, n (%)**				< 0.001
No	26074 (81.8)	18931 (83.5)	7143 (77.5)	
Yes	5817 (18.2)	3740 (16.5)	2077 (22.5)	
**CVD history,n(%)**				< 0.001
No	28487 (89.3)	20719 (91.4)	7768 (84.3)	
Yes	3404 (10.7)	1952 (8.6)	1452 (15.7)	
**Liver disease, n (%)**				< 0.001
No	30606 (96.0)	21866 (96.4)	8740 (94.8)	
Yes	1285 (4.0)	805 (3.6)	480 (5.2)	
**Cancer, n (%)**				< 0.001
No	28851 (90.5)	21041 (92.8)	7810 (84.7)	
Yes	3040 (9.5)	1630 (7.2)	1410 (15.3)	
**suicidal ideation, n (%)**				< 0.001
No	28469 (89.3)	20681 (91.2)	7788 (84.5)	
Yes	3422 (10.7)	1990 (8.8)	1432 (15.5)	
**Depression,n (%)**				< 0.001
No	29204 (91.6)	21276 (93.8)	7928 (86)	
Yes	2687 (8.4)	1395 (6.2)	1292 (14)	
**UI type, n (%)**				< 0.001
No	22671 (71.1)	22671 (100)	0 (0)	
SUI	2565 (8.0)	0 (0)	2565 (27.8)	
UUI	2338 (7.3)	0 (0)	2338 (25.4)	
MUI	2571 (8.1)	0 (0)	2571 (27.9)	
Other	1746 (5.5)	0 (0)	1746 (18.9)	

Abbreviations: UI urinary incontinence, SUI stress UI, UUI urge UI, MUI mixed UI, PIR poverty income ratio, MET metabolic equivalent,BMI body mass index,CVD history Cardiovascular Disease history.

Note: N frequency of participants, Mean ± SD for continuous variables, *P* value was obtained through one-way analysis of variance for continuous variables and chi-square tests for categorical variables.

### The relationship between UI and suicidal ideation

Univariate logistic regression analysis revealed factors significantly associated with suicidal ideation, including age, sex, BMI, race and ethnicity (excluding Hispanic black), marital status, education level, poverty income ratio, current smoking status, alcohol intake (excluding moderate consumption), and comorbidities such as hypertension, diabetes, arthritis, pulmonary disease, CVD history, and liver disease (*P* < 0.05), as shown in Table 2. Table 3 displays the multivariable logistic regression outcomes for the association between UI and suicidal ideation. The initial unadjusted model (Model I) revealed that a heightened risk of suicidal ideation was associated with UI (OR: 1.91, 95% CI: 1.78–2.06; *P* < 0.001). This association persisted even after we adjusted for demographic and health-related variables in Model II (OR: 2.02, 95% CI: 1.85–2.21, *P* < 0.001) and remained significant after further adjustment for chronic conditions in Model III (OR: 1.9, 95% CI: 1.74–2.08, *P* < 0.001). The inclusion of depression in Model IV did not significantly alter the results (OR: 1.54, 95% CI: 1.39–1.7, *P* < 0.001). Among the UI types, MUI participants had the highest odds of suicidal ideation. Compared to those without UI, the odds of suicidal ideation were significantly greater for individuals with MUI (OR: 2.11, 95% CI: 1.83–2.44; *P* < 0.001), SUI (OR: 1.4, 95% CI: 1.19–1.65; *P* < 0.001), and UUI (OR: 1.37, 95% CI: 1.16–1.62; *P* < 0.001) after comprehensive adjustments.

**Table 2:** Univariable regression analysis.

Variable	OR(95%CI)	P-value
Age	1 (0.99~1)	0.001
Sex:Female vs. Male	1.28 (1.19~1.37)	<0.001
BMI	1.02 (1.01~1.02)	<0.001
Race/Ethnicity:ref. = Mexican American		
Hispanic Black	1.06 (0.96~1.17)	0.225
Hispanic White	1.25 (1.13~1.38)	<0.001
Other Hispanic	1.69 (1.51~1.9)	<0.001
Other	0.8 (0.7~0.92)	0.001
Marital Status:ref. = Married		
Never married	1.55 (1.41~1.7)	<0.001
living with a partner	1.78 (1.58~2.02)	<0.001
other	1.85 (1.69~2.01)	<0.001
PIR:ref.< = 1.3		
1.3–3.5	0.55 (0.51~0.6)	<0.001
>3.5	0.3 (0.27~0.33)	<0.001
Education level:ref. = Less than high school		
High school or equivalent	0.71 (0.64~0.78)	<0.001
Above high school	0.5 (0.46~0.54)	<0.001
Smoking status:ref. = Former		
Never	1.1 (1~1.2)	0.048
Current	2.21 (2.03~2.4)	<0.001
Alcohol intake:ref. = Former		
Heavy	1.4 (1.23~1.58)	<0.001
Mild	0.79 (0.7~0.89)	<0.001
Moderate	1.06 (0.93~1.21)	0.394
Never	1.4 (1.25~1.58)	<0.001
MET:<500 vs. > = 500	0.78 (0.73~0.84)	<0.001
Hypertension:Yes vs. No	1.29 (1.2~1.38)	<0.001
Diabetes:Yes vs. No	1.36 (1.25~1.48)	<0.001
Arthritis:Yes vs. No	1.87 (1.73~2.01)	<0.001
Pulmonary disease:Yes vs. No	1.87 (1.72~2.02)	<0.001
CVD history: Yes vs. No	1.94 (1.76~2.14)	<0.001
Liver disease:Yes vs. No	2.09 (1.81~2.41)	<0.001
Cancer:Yes vs. No	1.08 (0.96~1.22)	0.2
Depression:Yes vs.No	22.26 (20.34~24.37)	<0.001
UI:Yes vs.No	1.91 (1.78~2.06)	<0.001
UI type:ref. = No		
SUI	1.51 (1.33~1.71)	<0.001
UUI	1.52 (1.34~1.74)	<0.001
MUI	3.28 (2.96~3.63)	<0.001
Other	1.28 (1.09~1.49)	0.002

**Table 3:** Multivariable regression analysis of the association between urinary incontinence and suicidal ideation.

Variable	Model I		Model II		Model III		Model IV	
	OR(95%CI)	P-value	OR(95%CI)	P-value	OR(95%CI)	P-value	OR(95%CI)	P-value
**UI**								
No	Ref							
Yes	1.91 (1.78~2.06)	<0.001	2.02 (1.85~2.21)	<0.001	1.9 (1.74~2.08)	<0.001	1.54 (1.39~1.7)	<0.001
**UI type**								
No	Ref		Ref		Ref		Ref	
SUI	1.51 (1.33~1.71)	<0.001	1.72 (1.49~1.98)	<0.001	1.64 (1.42~1.89)	<0.001	1.4 (1.19~1.65)	<0.001
UUI	1.52 (1.34~1.74)	<0.001	1.75 (1.51~2.02)	<0.001	1.66 (1.43~1.92)	<0.001	1.37 (1.16~1.62)	<0.001
MUI	3.28 (2.96~3.63)	<0.001	3.31 (2.93~3.74)	<0.001	2.96 (2.61~3.35)	<0.001	2.11 (1.83~2.44)	<0.001
Other	1.28 (1.09~1.49)	0.002	1.45 (1.23~1.72)	<0.001	1.42 (1.2~1.69)	<0.001	1.28 (1.06~1.54)	0.011
P **for trend**		<0.001		<0.001		<0.001		<0.001

Model I:no adjusted.

Model II:age, sex, marital status, race/ethnicity, education level, poverty income ratio,BMI,smoking status, alcohol intake, MET.

Model III:Model II+hypertension, diabetes, CVD history, arthritis, pulmonary disease,liver disease, cancer.

Model IV:Model III+depression.

### Subgroup analysis

**Fig 2:**
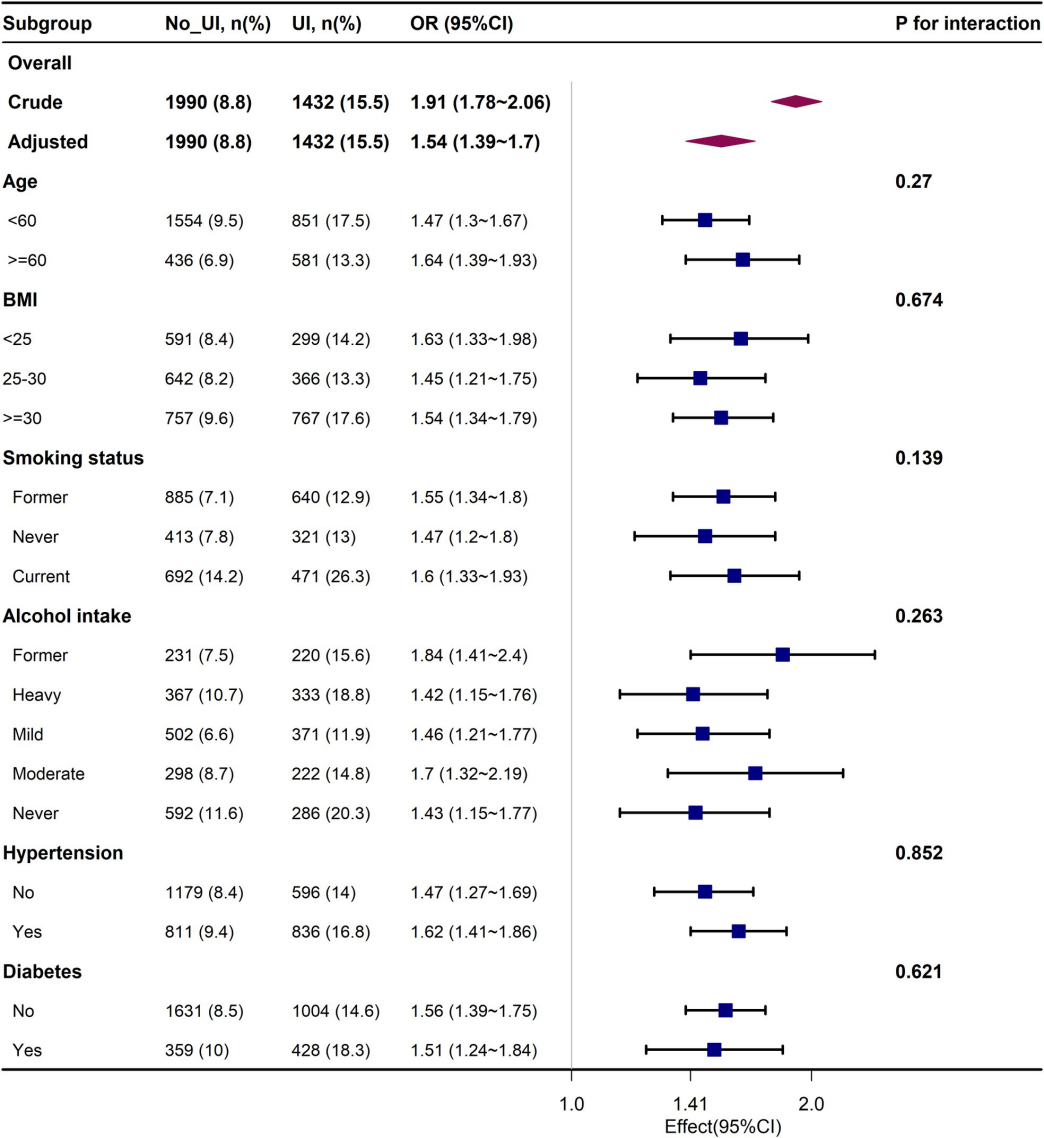
The relationship between UI and suicidal ideation according to basic features.

Fig 2 shows the results of subgroup analyses, considering factors such as age, sex, BMI, somking status, alcohol intake and prevalent comorbidities such as hypertension and diabetes, no statistically significant interactions were revealed (all *P* > 0.05). In all subgroups, there was a positive correlation between urinary incontinence and suicidal ideation.

### Sensitivity analyses

The robustness of the findings was confirmed in sensitivity analyses, where imputing missing covariates had a negligible impact on the results. Even after comprehensive adjustment for all covariates, the results retained statistical significance (OR: 1.53, 95% CI: 1.4–1.68, *P* < 0.001).

## Discussion

This study has yielded significant findings, shedding light on the relationship between UI and suicidal ideation. To summarize, our findings suggest two key points. First, individuals with UI are more prone to experiencing thoughts of self-harm when compared to those without UI. Second, this association persists, irrespective of baseline characteristics and other comorbid conditions, including depression.

To provide context, prior research has established that UI can lead to increased stress, anxiety, and depression [36]. Additionally, emotional issues can contribute to the occurrence of UI [37]. Although there is limited research on the relationship between urinary incontinence and suicidal ideation, some significant findings have been reported. For example, a cross-sectional study conducted in Ethiopia revealed that among untreated obstetric fistula patients [38], a striking 97.4% (38 out of 39) suffered from urinary incontinence, with 92.3% (36 out of 39) experiencing depression, and over 50% reported having suicidal ideation. These results suggest a potential correlation between urinary incontinence and suicidal thoughts. Moreover, a separate study in Uganda found that [13] although the quality of life was significantly impacted in patients who had undergone obstetric fistula repair surgery (with a Consultation on Incontinence Questionnaire-Quality of Life score of 62.77±12.76, ranging from 28 to 76, and a median score of 67), there was no statistically significant difference in the occurrence of suicidal ideation compared to patients without urinary incontinence.The differences in conclusions maybe due to varying sample sizes in studies. However, our findings are based on a larger, more diverse U.S. population dataset. By including additional covariates in our analysis, we aimed to enhance understanding of the significant link between urinary incontinence and suicidal ideation.

Urinary incontinence patients frequently face a range of challenges, including embarrassment, decreased well-being, financial burdens, daily functional impairments, social phobia, loneliness, and diminished self-esteem [12]. Loneliness plays a pivotal role, negatively impacting self-esteem and contributing to social phobia, isolation, and strained social relationships. An additional study demonstrated that elderly individuals with disharmonious social relationships were 57% more likely to have suicidal tendencies [39]. These issues collectively contribute to suicidal thoughts among UI patients. Moreover, a related cross-sectional study confirmed a strong connection between the decline in well-being over the past 12 months and suicidal ideation (OR: 0.81, 95% CI: 0.79–0.83) (33). Economic challenges and barriers to daily activities further exacerbate the burden on urinary incontinence patients. It is important to note that self-perceived burden (perceiving oneself as a burden) and perceived burden on others (being seen as a burden by others) have been identified as potential risk factors for suicidal tendencies [40].

Mental illnesses, particularly anxiety and depression, are recognized as significant factors that increase the risk of suicidal ideation [41,42]. UI is notably associated with depression [43]. However, the impact of depression as a crucial covariate on the strength of the relationship between urinary incontinence and suicidal ideation remains uncertain. After adjusting for depression factors in this study (Model IV), the association between urinary incontinence and suicidal ideation still holds statistical significance (OR: 1.54, 95% CI: 1.39–1.7, *P* < 0.001). However, the adjusted association strength of depression is significantly lower than in the unadjusted model (Model III) (OR: 1.9, 95% CI: 1.74–2.08, *P* < 0.001). These findings suggest that while depression may influence the strength of the relationship between UI and suicidal ideation, it does not negate the overall significance of this association.

The association between urinary incontinence and suicidal ideation is likely mediated by serotonin, a crucial neurotransmitter implicated in bladder function [43,44]. Furthermore, heightened sympathetic nervous system activity linked to suicide and depression may elevate levels of cortisol and catecholamines in circulation, potentially inducing physiological alterations in the bladder and consequently, urinary incontinence [44–46].

Our study has several strengths.Firstly, we utilized data from the NHANES database, ensuring a representative sample size. Secondly, we meticulously adjusted for covariates, such as age, sex, race, and comorbidities, and evaluated their stability across various statistical models. Furthermore, sensitivity analyses, including multiple imputation of missing data, were conducted to validate the robustness of our findings. However, it is essential to acknowledge certain limitations. Primarily, our reliance on questionnaires and self-reports for defining variables like suicidal ideation and urinary incontinence may introduce recall bias and subjective bias. While NHANES employs a multistage stratified probability design across 15 regions, reducing the likelihood of resampling the same participants in different cycles. Additionally, while we adhered to the established definition of suicidal ideation based on the PHQ-9, which primarily serves as an initial screening tool for suicide risk,it is noteworthy that the PHQ-9 may not capture non-fatal suicide, which may not be as sensitive or comprehensive as a dedicated suicide risk assessment tool [47,48], potentially underestimating the association between urinary incontinence and suicidal ideation.Moreover, the cross-sectional nature of our study precludes causal inference, underscoring the necessity for further investigations to elucidate the complex relationships between these variables. Despite adjusting for numerous confounders, the presence of unmeasured confounders cannot be entirely ruled out.Given these constraints, future research endeavors should incorporate well-designed prospective studies, supplemented by qualitative research components, to effectively inform health policy and patient care decisions.

In conclusion, this study underscores a significant association between UI and suicidal ideation. Despite the profound impact of incontinence on productivity and quality of life, a significant number of patients forego treatment [49,50], potentially due to societal stigma and psychological barriers. Hence, there is a pressing need for concerted action from healthcare providers and patients alike. Patients experiencing urinary incontinence should actively seek support from a multidisciplinary team of healthcare professionals. Additionally, physicians must prioritize addressing mental health concerns in patient encounters, while routine screening for associated psychiatric disorders can aid in early identification of individuals at heightened risk [51]. Collaborative efforts with mental health specialists can enhance the efficacy of interventions, thereby optimizing therapeutic outcomes [52,53].
